# Emerging Nutritional Interventions for Age-Associated Cellular Decline

**DOI:** 10.1093/geroni/igaa057.2761

**Published:** 2020-12-16

**Authors:** Anurag Singh

**Affiliations:** Amazentis SA, Lausanne, Vaud, Switzerland

## Abstract

Aging is associated with a progressive decline in cellular health leading to dysfunction in organs with a high metabolic demand. A key feature of age associated cellular decline is impairment of mitochondrial quality control pathways such as mitophagy. Deficits in optimal functioning of these pathways results in a compromise in cellular bioenergetics that ultimately leads to mitochondrial dysfunction. Promising nutritional interventions have emerged that boost mitochondrial health such as nicotinamide riboside (vitamin B3 precursor) and Urolithin A (gut metabolite of compounds found in pomegranates), that act via different mechanisms of action to improve overall mitochondrial health. Recent literature on the evidence behind these interventions will be presented and discussed during this symposium. We will also share recent clinical evidence from double-blind placebo-controlled studies with Urolithin A. Our results suggest that nutritional interventions such as Urolithin A are promising approaches that can be employed to manage age associated cellular decline.

